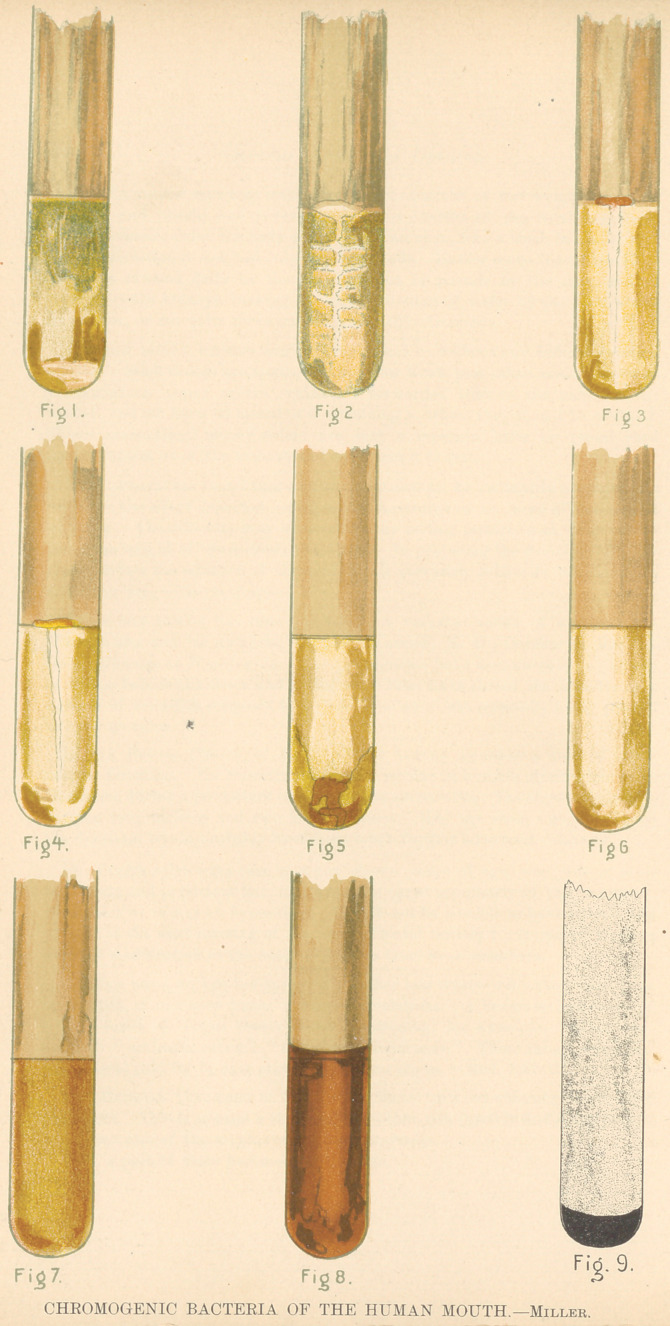# Current News and Opinion

**Published:** 1888-07

**Authors:** 


### Current News and Opinion.

### A NEW DENTAL SYRINGE.

BY M. G. JENISON, M. D., D. D. S., MINNEAPOLIS, MINN.

Recently there have been several suggestions in the dental journals as to the best method of keeping syringes in order. When they are not so we all know the annoyance that results, such as a dry piston that requires a long time to put in operation, and leaky packing that allows the medicine to drop on the operator's hands or upon the patient. If a bulb syringe is used it soon breaks and leaks, but the almost absolute necessity for some instrument of this description is met with every day in any full practice. In abscesses, necrosis, pyorrhœa, etc., the remedial agent must be brought in contact with the diseased part to produce the best result, and as in many cases we wish to employ two or more remedies so is the annoyance of some forms of syringe increased by the time and trouble required in changing and cleansing.

My Syringe, as shown in the drawing, is different in principle and application from any I have seen for dental purposes, and to me has been the most satisfactory of the many I have tried. As will be seen, no piston is employed, the air pressure or cushion taking the place of it, and that does not become dry and fail to work. The bulb used to apply the force can be as easily controlled as any piston, and is so situated that the medicines do not enter it, the air entering the bottle at the top, forcing the remedies down and through the tube, starting at the bottom. If any of the air should pass entirely through to the seat of the disease, which is not likely, it first passes through the medicine, rendering it harmless.

The screw-topped bottle admits of rapid changes and thorough cleansing. The bulb and attachment are of rubber, and the tube leading from the bottle is of metal. Platina is probably best for this, though not necessary, but whatever is employed should end in a fine gold point. In the use of this syringe, as with some others, an assistant is an advantage, but not a necessity. The force can be varied from that which will carry only a few drops through the point of the tube to that which is strong enough to force a remedy through any fistulous tract, or to thoroughly cleanse any sized sac that we are liable to meet with.

All that I claim in this is a new adaptation of established principles, and one that has given me complete satisfaction where pathological conditions requiring this form of treatment exist.

### GAS FURNACES, ENAMEL FILLINGS, ETC.

A REPLY TO DR. WILLIAM HERBERT ROLLINS' ARTICLE IN THE JUNE NUMBER OF THE INDEPENDENT PRACTITIONER.

BY C. H. LAND.

The cases Dr. Rollins has cited have no relation to my improvements, which he openly confesses in the sentence in which he admits that I am entitled to the use of nitrogen and the arrangements of parts. We want no better evidence of his entire ignorance of the valuable features of these inventions. The mere assertion that he is entitled to the original discovery is no evidence of the facts. My greatest surprise is that in one instance he claims to have injected hot air into the muffle and then indirectly admits that it was in the combustion chamber, and in another sentence he openly declares that I originally produced a separate means for the purpose of injecting nitrogen into the muffle. Did he realize that the atmosphere could pass through this same separate arrangement of parts? And when he supplements his remarks with the assertion that he could not comprehend why this was so constructed, did he realize that this difference was one of the most valuable parts of my invention? Such testimony is all the evidence needed to vindicate my entire rights.

Since my furnace has been on the market for upwards of four years and in use in nearly every State in the Union, also in foreign countries, and it was on exhibition before the American Dental Association in August, 1886, demonstrating my system of restoring and filling teeth with porcelain, also at various dental societies years previous to this, is it not surprising that he has only just discovered that such things are actually successful? In the references cited he has not even thought of or referred to the fixed gas, C O. He merely asserts that gasing is due to certain component parts of the material being reduced. If he would like to know just what the reaction is, I can accommodate him, and as he seems to be somewhat familiar with school-boy notions, I will be glad to receive and welcome all those who have not out-grown their youth.

I can produce witnesses who assisted me in my experiments with furnaces and porcelain many years previous to any dates that Dr. Rollins has mentioned. I can show by the files in the Patent Office that my furnace was on record one year previous to the cases he has cited. I will also produce several witnesses who can testify to my experiments with nitrous oxide gas and coal gas in the blow-pipe, as also pure oxygen and coal gas, oxygen and hydrogen, and the effects of nitrogen in combination with them. All these, including the injection of the atmosphere into the muffle, were successfully applied to coal furnaces anterior to the production of any gas furnace. In conclusion, as Dr. Rollins has intimated that he does not care to refer to this matter again, perhaps it will be just as well to let it rest here.

---

### IS DENTISTRY A LIBERAL PROFESSION?

When the American Medical Association instituted a section on dentistry, it recognized to a degree the claim of some dentists that their art is properly one of the legitimate specialties of medicine. While it is unquestionably true that dentistry should occupy this position in medicine, it is just as certainly true that at the present it does not. The education in dental schools is not that given to doctors of medicine; the restrictions of dental practice, and the legal requirements of dentists, are not those that are applied to physicians, and especially does the true professional sentiment, the *esprit de corps*, seem to be wofully lacking among our brethren of the forceps and drill. If dentistry would lay claim to the prerogatives of a liberal profession, it must first establish the justice of the claim, and this cannot be done at present.

One of the most noticeable derelictions of dentistry, one that is neither creditable nor humane, is the general lack of charitable work by the fraternity. If dentistry is only a luxury that those who pay can have, but that the poor can very well do without, there is no need for gratuitous work, but no dentist will admit that his vocation is anything but a necessity to the people. If, then, the views of the dentist are accepted as true, we have an immense population with an urgent need which cannot be gratified, because there is no provision for charitable work. In Allegheny county, with a population of half a million people, with charitable organizations covering nearly the whole field of human necessities, the dental profession alone withholds its gratuitous services. Doubtless there are benevolent individuals in the guild who give their skill sometimes to suffering poverty, but this is not enough; it is in such cases a personal, and not a professional benevolence. What is needed is an organized charity, a dental dispensary for the poor. This will in itself do much to lift dentistry out of the realms of trade, and clothe it with the dignity of a liberal profession.—*Pittsburgh Medical Review.*

It is sometimes well to view ourselves as others see us, and to obtain a lesson from our critics. For this reason we give place to the above at the request of a dental friend.—EDITOR.

---

### AMERICAN DENTAL ASSOCIATION.
### SOUTHERN DENTAL ASSOCIATION.

A joint meeting of the two Associations will be held in Louisville, Ky., commencing August 28, 1888. This will be the 28th annual meeting of the American and the 20th of the Southern Society, and for the sessions the committees of the two have provided the following programme:

Tuesday Morning, 9 a. m.—Separate meetings of the two Associations for the payment of dues, the receiving of credentials and the transaction of routine business.

Tuesday Afternoon, 2.30 p. m.—Meeting of the different joint committees for the examination of the papers to be read by them.

Tuesday Evening, 7.30 p. m.—(As the joint session is only for scientific and social purposes, nothing but professional subjects will be discussed or acted upon). Meeting of the joint session, to be presided over by Frank Abbott and B. H. Catching President Abbott's address. President Catching's address. Discussion on the same. Reports of joint committees and discussions thereon. Announcements from the Chair. Adjournment.

Wednesday Morning, 9 a. m.—Meeting of the joint session, presided over by B. H. Catching.

Wednesday Afternoon —To be devoted to the work of the joint committees, or to business meetings of the Associations if such should be necessary, or to clinics

Wednesday Evening, 7.30 p. m.—Meeting of joint session, presided over by Frank Abbott.

Thursday Morning, 9 a. m.—Meeting of joint session, presided over by B. H. Catching.

Thursday Afternoon.—Committee work, or business meetings, or clinics.

Thursday Evening, 7.30 p. m.—Meeting of joint session, presided over by Frank Abbott.

Friday Morning, 9 a. m.—Meeting of the joint session, presided over by B. H. Catching.

Friday Afternoon, 3 p. m.—Separate meetings of the two Associations for the selection of place of next meeting, the election of officers, and the transaction of such other business as may come before them.

Friday Evening, 7.30 p m.—Meeting of joint session, presided over by Frank Abbott

Saturday Morning, 8.30 a. m. to 1.30 p. m.—Clinics.

Saturday Afternoon, 3 p. m.—Separate meeting of the two Associations for closing business.

The joint committees will be called in the following order, and any committee failing to respond will be passed and not again called until all the others have been.

The reports of these committees will be written and offered by the chairmen, and will specify the papers to be presented and the order in which they shall be read, and such subjects for discussion or such suggestions as they may wish to bring to the attention of the joint session.

Operative Dentistry.—Geo. H. Winkler, of the Southern Dental Association; E. T. Darby, of the American Dental Association, Chairmen. Report to be presented by Geo. H. Winkler.

HISTOLOGY AND MICROSCOPY.—Frank Abbott, of the American Dental Association; John G. McCullock, of the Southern Dental Association, Chairmen. Report by Frank Abbott.

MATERIA MEDICA AND THERAPEUTICS.—John C Story, of the Southern Dental Association; A. W. Harlan, of the American Dental Association, Chairmen. Report by John C. Story.

PHYSIOLOGY AND ETIOLOGY.—H. A Smith, of the American Dental Association; E S. Chisholm, of the Southern Dental Association, Chairmen. Report by H. A. Smith.

ANATOMY, PATHOLOGY AND SURGERY.—Morgan Adams, of the Southern Dental Association; T. W. Brophy, of the American Dental Association, Chairmen. Report by Morgan Adams.

PROSTHETIC DENTISTRY, METALLURGY AND CHEMISTRY.—J. Rollo Knapp, of the American Dental Association; V. E. Turner, of the Southern Dental Association, Chairmen. Report by J. Rollo Knapp.

DENTAL EDUCATION, LITERATURE AND NOMENCLATURE.—J. Taft, of the Southern Dental Association; W. H Atkinson, of the American Dental Association, Chairmen. Report by J. Taft.

HYGIENE.—Geo. J. Friedrichs, of the American Dental Association; J. L. Mewburn, of the Southern Dental Association, Chairmen. Report by George J. Friedrichs.

COMMITTEE ON VOLUNTARY PAPERS.—G. F. S Wright, of the Southern Dental Association; S. H Guilford, of the American Dental Association, Chairmen.

This committee will examine all papers not previously presented to the other committees, and those that are accepted they will refer to the appropriate committees.

All papers to be read before the joint session, except the two Presidents' addresses, must be placed in the hands of the chairmen of the appropriate joint committees who will examine them and report those only they deem worthy of presentation to the joint session

The following rules of order will govern this joint session:

No member of either Association shall be entitled to the floor unless he is in good standing and his dues are fully paid.

No person shall speak more than twice upon the same subject nor more than ten minutes in all, unless consent is given by a majority vote of the joint session.

No one shall be permitted to address the meeting before giving his name and residence, which shall be distinctly announced from the chair.

When a paper has been read it shall at once be handed to the Secretary of the Association from which it came.

Any paper or report to be entitled to publication in the transactions must be placed in the hands of the Publication Committee by the 15th of September, 1888, and must be so prepared that the proof-sheets furnished the author shall be returned to the committee without material alteration or addition.

Roberts' Rules of Order shall be the authority governing this meeting, if any is needed more than is embodied in the foregoing rules.

### CONNECTICUT VALLEY AND MASSACHUSETTS DENTAL SOCIETIES.

The Connecticut Valley Dental Society and the Massachusetts Dental Society will hold a Union Meeting in Boston on the 10th, 11th, 12th and 13th of July next, at the Institute of Technology.

All the dental societies in New England will be invited to unite with them, so that the meeting promises to be the largest ever held in this part of the country.

Programmes can be obtained upon application to the Secretary of either Society.

G. F. Eames, M. D., D. D. S., 62 Trinity Terrace, Boston, Mass.,
Secretary Mass. Dental Society.

Geo. A. Maxfield, D. D. S., Holyoke, Mass.,
Secretary Conn. Valley Dental Society.

---

### NEW JERSEY STATE DENTAL SOCIETY.

The eighteenth annual session of the New Jersey State Dental Society will convene at the West End Hotel, Asbury Park, Wednesday, July 18, 1888, at 10 o'clock, A. M., and continue in session until final adjournment. A large number of valuable papers are promised by men of professional eminence, and important clinics in practical work will be given. An unusually large and profitable meeting is confidently anticipated.

Chas. A. Meeker, Secretary,
27 Fulton Street, Newark.

---

### DENTAL CONVENTION.

A meeting of the members of the Dental Profession in the Province of Ontario will be held in the College of Dentistry, in Toronto, on Tuesday, July 17th, at three o'clock, P. M., for the formation of a Dental Society. The meeting will be continued on the following day, when interesting papers will be contributed by prominent members of the profession in the United States and Canada. All American Dentists are cordially invited to be present on the occasion.

By order,
Geo. C. Davis, Secretary, pro tem.

---

### WISCONSIN STATE DENTAL SOCIETY.

The eighteenth annual meeting of the Wisconsin State Dental Society will be held in Milwaukee, commencing Tuesday, July 17th, and continuing three days. A good number of papers upon dental subjects are promised, and clinics will be given by prominent members of the profession. W. S. Sullivan, Sec.

---

### MISSOURI STATE DENTAL ASSOCIATION.

The Missouri State Dental Association will hold its twenty-fourth annual meeting at Pertle Springs, Warrensburg, Mo., July 10, 11, 12 and 13, 1888.

An attractive programme has been provided, and members of the profession are cordially invited to be present. William Conrad, Cor. Sec.

Dr. W. H. Taggart, of Freeport, Ill., has devised an implement which should be in the possession of every operative dentist It is a Corundum Point and Disk Maker, and for simplicity of construction and effectiveness it is all that could be desired. Moulds for the different forms accompany it, and any office boy or girl can turn out the most perfect corundum points at the rate of twenty or thirty an hour without help from the dentist. We have been using them for some time with great satisfaction. Their cost is merely nominal, and provided with the machine the dentist can use them as freely and with as little regard for economy as though they grew spontaneously, for the supply will be inexhaustible. The forms supplied are admirable, and the points and disks can be made from any grade of corundum, old lathe wheels being utilized in their manufacture. Blank mandrels can easily be made from Stub's wire, gauge No. 42, or they can be purchased at a small expense, and a supply once obtained, they can be used again and again for an indefinite time. We can most heartily commend the machine as an economical investment for every operative dentist, to say nothing of the convenience of a never-ending supply of one of the essentials for good work.

---

The further extension of the dental college term is talked of in some quarters, which reminds us of a remark made by one of the most experienced teachers in the profession. He had been succeeded in a well-established school by a man of his own teaching, and in answer to our question as to how his successor would get on, said: "He'll have trouble to get through his term" "How so?" "He'll tell all there is of it in less than three months My own trouble consisted in finding something to say that I had not already said, and at the same time avoid letting the students know that I had not overdrawn my account." "Is that why you bombarded us with two lectures on 'Epochs in Dentistry'?" "Just so." More work and less talk would come nearer meeting the average student's requirements.—*Dental Exchange.*

Hum! Let us see. A three months' course means thirteen, or twenty-six hours of instruction, according to whether the professor in question lectured once or twice a week If he lectured three times, either the faculty was a small one or some of the rest were cut short. Twenty-six hours! And he overdrew his account—told all there was in his department in a three-months' course—exhausted dentistry in thirteen weeks. Well, we should say that it was high time that he was superseded, and a man was found whose pond was not so soon pumped dry.

---

The editor of *The British Journal of Dental Science* is peculiarly unfortunate. He seems to be possessed by a kind of American-dentist-phobia, and members of the obnoxious class will persist in visiting and even settling in London and other English cities; what is worse, they seem to get on very well, and what is worst, Englishmen and English newspapers *will* magnify American dentistry The respected editor struggles manfully with the situation, but the tide seems to be against him. We can assure him that there are very respectable men and fairly skillful operators among American dentists, and when they know a thing they like to have all the rest of the profession learn it too To be sure, there are far too many quacks and unprincipled adventurers, but we have a faint recollection of seeing very vigorous denunciations of certain English dentists in the pages of our good contemporary, and this has led us to suspect that even our English brethren are not unvexed by a like class

P. S.—We see by the last number that a change has come o'er the spirit of his dream, and our respected contemporary is now "booming" Dr. E. Parmly Brown, who is giving clinics in London, for all he is worth.

---

THE CHAIRMAN of Section VI of the American Dental Association has sent to the members the following circular. We need not say that answers will be welcomed from any reputable source:

DEAR DOCTOR:—Section VI of the American Dental Association (Physiology and Etiology) desires to obtain more exact information regarding *implantation* of teeth. Will you please aid in the preparation of the report to be made at the annual meeting at Louisville, by answering the enclosed questions and returning the paper promptly to H. A. SMITH, Chairman,
128 Garfield Place, Cincinnati, O.

I. What proportion of your cases of *Implantation* do you regard as successful?

II. If any have failed, what was the cause of such failure?

III. Do implanted teeth assume the color of natural teeth in the mouth?

IV. What do you regard as the mode of attachment, if any?

---

DR. ROBERT BOXALL, in the *British Medical Journal*, says that corrosive sublimate (merc. chl.) and iodine are incompatible. A small addition of the former fixes the free iodine, as may be seen by the immediate bleaching of the iodine solution. Carbolic acid and iodine are also incompatible An exceedingly small admixture with carbolic acid is sufficient to fix the whole of the free iodine. Consequently the two should never be compounded together, and such preparations as carbolate of iodine are of no use. Corrosive sublimate and soap are incompatible, as the latter will throw down the mercury. Carbolic acid is made inert by olive oil, the latter fixing the phenol. Koch has shown that bacillus spores are capable of living and developing after having been immersed in carbolized oil for four months

---

REV. W. H. DALLINGER, a well-known microscopist and observer of England, has been experimenting with micro-organisms to determine their power of adapting themselves to changed conditions. He made cultivations of certain organisms at ordinary air temperature, and then gradually increased it at the rate of one degree a month, continuing the cultivations. At 73° F. he reached a point where he was obliged to stop and let the temperature fall a little, as the organisms had reached the limit of their endurance. After two months the temperature was again raised, but at 78° he was again obliged to halt until the succesive colonies had become acclimated. At 93° he was obliged to wait nine months, and so successive critical points were found at 107°, 147° and 158°, when an accident destroyed the cultivations and put an end to the experiment.

Dr. Whitelaw says that water forms three-fourths of the weight of living animals and plants, and covers about three-fourths of the earth's surface. Prof. Chaussier dried the body of a man in an oven, like a brick in a kiln, and after desiccation it weighed only twelve pounds. Rather more than a pound of water is exhaled daily by the breath, about 1¾ pounds by the skin, and 2¾ pounds by the kidneys, making the daily emissions of water by the body about 5½ pounds, or not quite three quarts.—*Scientific American.*

---

Foreign money orders are sometimes sent us without any definite separate advices Such orders are stopped at the New York post-office and exchanged for American money orders, which are forwarded without the name of the sender, and we have no means of determining to whom they should be credited. When international money orders are sent a separate letter or postal card should inform us of the amount and by whom it is sent.

---

Dr Laplace has found that antiseptic dressings, as ordinarily prepared, do not have the effect expected, owing to the formation of an inert albuminate of mercury. The difficulty may be overcome by adding tartaric acid, four or five times the weight of the corrosive sublimate. In preparing the dressings Dr. Laplace advises the addition of the acid to all corrosive sublimate solutions for antiseptic use.—*Pharmaceutical Era*

---

*Forest and Stream* has been publishing a valuable series of articles upon "Snake-bite and its Antidote," by H. C. Yarrow, M. D., Curator in the National Museum at Washington. The experiments have demonstrated a number of things before unknown, and at the same time have shown the inutility of a number of remedies supposed to be infallible, ammonia especially being proved to have no value.

---

In the *Boletin Clinico*, of Lerida, Senor Lorens mentions a case of intra-uterine dentition. He recently attended a woman in humble circumstances in Barcelona during a permature confinement at six months. The child had already cut the four incisors and two lower canines. Had the woman gone full time the dentition would probably have been much further advanced.—*Lancet.*

---

Dr. Voisin relates the case of a girl eleven years of age, who was a most inveterate and persistent liar, and whom he cured completely of this reprehensible habit by means of hypnotism. Will it not be possible to have a hypnotist present at the next meeting of the ——— Dental Society? It might work a decided modification in the reports of cases from some members.

---

German drug stores are apparently rather less trustworthy than American or English, if one may judge of recent revelations. A Berlin society sent out a long series of bogus prescriptions, containing, for example, "tuber cinereum," "urticaria rubra," "pemphygus foliaceous." These things were dispensed and paid for in over sixty Berlin drug stores —*Med. Record.*

---

The Medical Colleges of the United States turn out annually about 4,000 graduates. This is at least a thousand more than can possibly find employment. What becomes of the surplus?—*Medical Exchange.*

Well, a part of them become poor dentists.

## Figures and Tables

**Figure f1:**
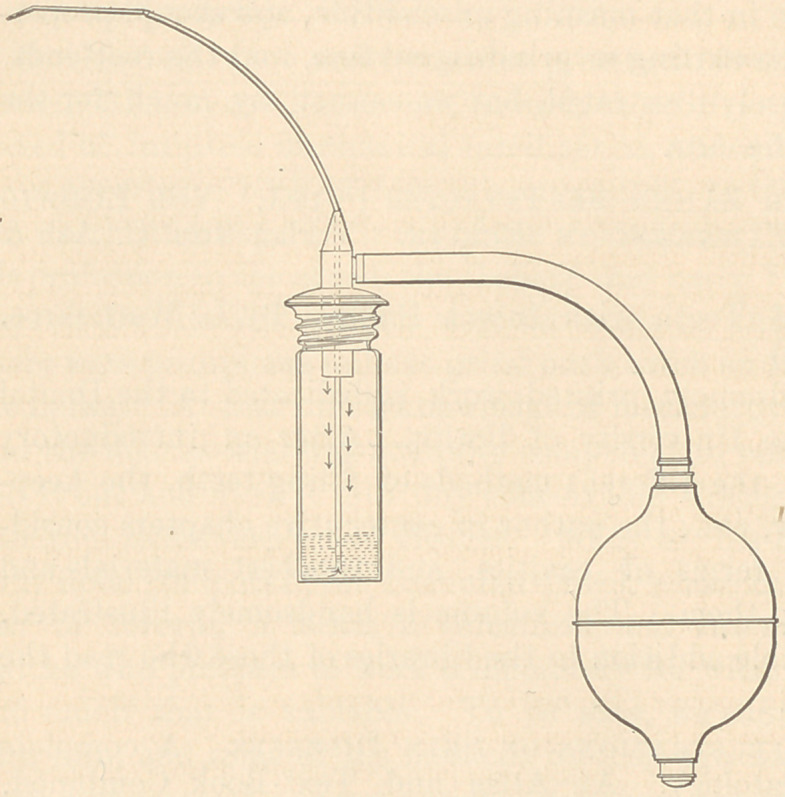


**Figure f2:**